# Impact of non-alcoholic fatty liver disease and smoking on colorectal polyps

**DOI:** 10.18632/oncotarget.20462

**Published:** 2017-08-24

**Authors:** Qin-Fen Chen, Xiao-Dong Zhou, Dan-Hong Fang, Yang-Jie Sun, Qian Zhao, Jun-Hua Huang, Yin Jin, Jian-Sheng Wu

**Affiliations:** ^1^ Department of Gastroenterology, First Affiliated Hospital of Wenzhou Medical University, Wenzhou 325000, China; ^2^ Department of Cardiovascular Medicine, First Affiliated Hospital of Wenzhou Medical University, Wenzhou 325000, China; ^3^ Medical and Health Care Center, First Affiliated Hospital of Wenzhou Medical University, Wenzhou 325000, China

**Keywords:** colorectal polyps, smoking, non-alcoholic fatty liver disease, health check

## Abstract

**Objectives:**

Non-alcoholic fatty liver disease (NAFLD) and smoking have similar mechanisms of promoting colorectal polyps. The potential link between NAFLD and smoking in men and colorectal polyps has not been adequately evaluated. The aim is to investigate this association.

**Methods:**

A retrospective cross-sectional study was conducted on 2409 individuals undergoing a health check. Univariate and multivariate logistic regression were performed for analyzing the association between risk factors and colorectal polyps. Individuals were divided into four groups: Q1: NAFLD (-)/smoking (-); Q2: NAFLD (+)/smoking (-); Q3: NAFLD (-)/smoking (+); Q4: NAFLD (+)/smoking (+). Logistic analyses were used to explore associations for the whole study population and stratified groups.

**Results:**

The prevalence of colorectal polyps was 38.8% in males, and that of colorectal polyps in smokers and individuals with NAFLD were 47.0% (428/911) and 42.9% (267/622), respectively. With Q1 as reference, subjects with NAFLD (+) and smoking habits (+) had the highest ORs for colorectal polyps (OR = 2.64, 95% CI: 1.91 - 3.64, P < 0.001), adenomatous polyps (OR = 2.06, 95% CI: 1.38 - 3.05, P < 0.05), non-adenomatous polyps (OR = 1.97, 95% CI: 1.39 - 2.80, P < 0.05), ≥ 3 polyps (OR = 2.05, 95% CI: 1.31 - 3.22, P < 0.05) and proximal polyps (OR = 1.58, 95% CI: 1.02 - 2.45, P < 0.05) after adjusting for confounding variables.

**Conclusions:**

Men with NAFLD and smoking habits have an increasing risk of colorectal polyps.

## INTRODUCTION

Colorectal cancer is regarded as one of the most common human malignancies and the second leading cause of cancer-related death worldwide [1–3]. Colon polyps may transform into carcinoma due to genetic mutations [4]. The incidence of colorectal polyps has been rising dramatically over the past decades [5]. Colonoscopy and polypectomy are considered efficient methods to detect colorectal polyps and decrease the incidence of cancer [6]. Accumulating evidence has demonstrated that common risk factors, such as male gender, age, family history, metabolic syndrome (MS), insulin resistance, non-alcoholic fatty liver disease (NAFLD), smoking, alcohol consumption, diet and lifestyle contribute to the development of colorectal polyps [7–9].

Studies describing the mechanisms underlying the association between various risk factors and colorectal polyps are abundant and complex. The crucial role of insulin resistance and chronic inflammation in the occurrence and carcinogenesis of colorectal polyps has been reported in several experimental and observational studies [8, 10–13]. Bu-Tian Ji et al. demonstrated that smoking significantly increases the risk of both hyperplasic and adenomatous polyps [14]. The possible mechanisms by which colorectal polyps arise in individuals who smoke comprise insulin resistance, inflammation and oxidative stress [13, 15]. Moreover, NAFLD has been revealed as a risk factor for the prevalence of colorectal adenomatous polyps by several cohort studies [16, 17]. It is generally recognized that chronic inflammatory state and insulin resistance have crucial roles in the development of NAFLD [16]. Consequently, a similar mechanism may be involved behind the increase in the prevalence of colorectal polyps in the presence of NAFLD and smoking.

Men with NAFLD and smoking habits should be carefully monitored for the incidence of colorectal polyps. A cohort study by Claudia et al. reported that 1091 NAFLD patients enrolled in the non-alcoholic steatohepatitis clinical research network (NASH CRN) studies with liver biopsies exhibited accelerated progression of liver disease in the presence of smoking [18]. A recent cross-sectional study reported that smoking habit and fatty liver had a synergistic effect on MS, and the risk of MS increased by 9.5-fold in smokers with fatty livers relative to non-smoke without fatty livers [19]. NAFLD and smoking in men (NSM) may increase the prevalence of colorectal polyps. However, the association between NSM and colorectal polyps remains unknown. According to a cross-sectional survey of tobacco use among adults in China, which was carried out by the Global Adult Tobacco Survey (GATS), only 2.4% of women were smokers, whereas 52.9% of men were smokers [20].

Therefore, the primary focus of the present study was to explore the association between NSM and colorectal polyps and provide evidence for the early detection of colorectal polyps.

## RESULTS

### Subject characteristics

The demographics and clinical features of 2409 eligible participants are shown in Table 1. A total of 934 subjects were diagnosed with colorectal polyps. The prevalence of NAFLD in colorectal polyps group (n = 934) and polyp-free group (n = 1475) were 28.6% and 24.1%, respectively. The frequency of smoking was 45.8% in colorectal polyps group and 32.7% in polyp-free group. Subjects with polyps were older in age and had higher weight, BMI, SBP, DBP, FPG, AKP, γ-GT and higher frequency of alcohol use.

**Table 1:** Baseline characteristics between polyp-free group and colorectal polyps group in men

Variables	Polyp-free	Colorectal polyps	P-value
	n=1475 (61.8%)	n=934 (38.8%)	
**Age (years)**	44.65 ± 9.89	50.20 ± 9.70	<0.001
**Weight (kg)**	69.48 ± 10.18	70.87 ± 10.22	0.001
**Height (cm)**	169.13 ± 5.57	168.87 ± 5.81	0.272
**BMI (kg/m**^2^**)**	24.28 ± 3.22	24.81 ± 3.06	<0.001
**SBP (mmHg)**	127.29 ± 16.25	130.50 ± 17.85	<0.001
**DBP (mmHg)**	76.43 ± 11.75	78.16 ± 12.06	0.001
**FPG (mmol/L)**	4.86 ± 1.27	5.06 ± 1.49	<0.001
**TG (mmol/L)**	2.14 ± 2.05	2.15 ± 1.96	0.953
**TC (mmol/L)**	5.42 ± 1.16	5.42 ± 1.13	0.978
**HDL-C (mmol/L)**	1.22 ± 0.28	1.22 ± 0.29	0.864
**LDL-C (mmol/L)**	3.27 ± 0.86	3.26 ± 0.84	0.772
**ALT (IU/L)**	34.77 ± 33.71	35.19 ± 29.29	0.758
**AST (IU/L)**	29.03 ± 17.88	30.13 ± 18.98	0.153
**AKP (IU/L)**	77.48 ± 20.02	79.88 ± 24.41	0.013
**γ-GT (IU/L)**	57.61 ± 101.8	65.86 ± 79.17	0.037
**ALB (g/L)**	45.38 ± 3.16	44.85 ± 3.22	<0.001
**H. pylori n (%)**^a^	699 (50.1%)^a^	450 (50.9%)^a^	0.723
**Alcohol n (%)**	288 (19.5%)	260 (27.8%)	<0.001
**NAFLD n (%)**	355 (24.1%)	267 (28.6%)	0.015
**Smoking n (%)**	483 (32.7%)	428 (45.8%)	<0.001

BMI: body mass index; SBP: systolic blood pressure; DBP: diastolic blood pressure; FPG: fasting plasma glucose; TG: triglyceride; TC: total cholesterol; HDL-C: high density lipoprotein-cholesterol; LDL-C: low density lipoprotein-cholesterol; ALT: alanine aminotransferase; AST: aspartate aminotransferase; AKP: alkaline phosphatase; γ-GT: γ-Glutamyltransferase; ALB: albumin; H.pylori: helicobacter pylori; NAFLD: non-alcoholic fatty liver disease.

^a^: data are extracted from 2278 subjects.

### NAFLD and smoking are independent risk factors for colorectal polyps

Univariate and multivariate logistic regression analyses of the risk factors for colorectal polyps are presented in Table 2. Compared with the polyp-free group, the unadjusted ORs for colorectal polyps among variables were as follows: NAFLD (OR = 1.26, 95% CI: 1.05 - 1.52, P = 0.014) and smoking (OR = 1.74, 95% CI: 1.47 - 2.06, P < 0.001). All variables that were associated with colorectal polyps were identified as candidate variables in multivariate analysis. The results of multivariate analysis signified age (OR = 2.63, 95% CI: 2.19 - 3.15, P <0.001), smoking (OR = 1.83, 95% CI: 1.53 - 2.19, P < 0.001), NAFLD (OR = 1.33, 95% CI: 1.08 - 1.64, P = 0.007), obesity (OR = 1.15, 95% CI: 0.96 - 1.39, P = 0.130), hypertension (OR = 1.10, 95% CI: 0.90 - 1.34, P = 0.349), hyperglycemia (OR = 0.95, 95% CI: 0.70 - 1.29, P = 0.754) and alcohol use (OR = 1.28, 95% CI: 1.04 - 1.57, P = 0.020) after adjustment for age, smoking, NALFD, obesity, hypertension, hyperglycemia and alcohol use. The prevalence of colorectal polyps in smokers and individuals with NAFLD were 47.0% and 42.9%, respectively. Furthermore, significant association was found between NSM and colorectal polyps after controlling for possible confounders.

**Table 2:** Univariate and multivariate analysis of risk factors for colorectal polyps in men

	Polyp-free n=1475	Colorectal polyps n=934	Crude OR (95% CI)	P-value	Adjusted OR (95% CI)	P-value
**Age (year)**						
**< 50**	1046 (70.9%)	456 (48.8%)	1		1	
**≥50**	429 (29.1%)	478 (51.2%)	2.56 (2.16 - 3.03)	<0.001	2.63 (2.19 - 3.15)	<0.001
**Smoking**						
**No**	992 (67.3%)	506 (54.2%)	1		1	
**Yes**	483 (32.7%)	428 (45.8%)	1.74 (1.47 - 2.06)	<0.001	1.83 (1.53 - 2.19)	<0.001
**NAFLD**						
**No**	1120 (75.9%)	667 (71.4%)	1		1	
**Yes**	355 (24.1%)	267 (28.6%)	1.26 (1.05 - 1.52)	0.014	1.33 (1.08 - 1.64)	0.007
**Obesity**						
**No**	859 (58.3%)	491 (52.7%)	1		1	
**Yes**	614 (41.7%)	441 (47.3%)	1.26 (1.07 - 1.48)	0.007	1.15 (0.96 - 1.39)	0.130
**Hypertension**						
**No**	1104 (74.8%)	644 (69.0%)	1		1	
**Yes**	371 (25.2%)	289 (31.0%)	1.34 (1.11 - 1.60)	0.002	1.10 (0.90 - 1.34)	0.349
**Hyperglycemia**						
**No**	1335 (92.2%)	823 (89.7%)	1		1	
**Yes**	113 (7.8%)	95 (10.3%)	1.36 (1.02 - 1.82)	0.034	0.95 (0.70 - 1.29)	0.754
**Dyslipidemia**						
**No**	647 (44.9%)	396 (43.3%)	1			
**Yes**	795 (55.1%)	518 (56.7%)	1.07 (0.90 - 1.26)	0.463		
**Alcohol**						
**No**	1187 (80.5%)	674 (72.2%)	1		1	
**Yes**	288 (19.5%)	260 (27.8%)	1.59 (1.31 - 1.93)	<0.001	1.28 (1.04 - 1.57)	0.020

Adjusted for age, smoking, NALFD, obesity, hypertension, hyperglycemia and alcohol.

NAFLD: non-alcoholic fatty liver disease.

### The association between NSM and colorectal polyps

To obtain a deeper understanding of the association between NSM and colorectal polyps, individuals were divided into four groups (Q1, Q2, Q3 and Q4) according to the different statuses of NAFLD and smoking. The baseline characteristics of NAFLD and smoking status in men are shown in Supplementary Table 1. The prevalence for colorectal polyps was 32.1%, 38.3%, 45.6%, and 51.4% for individuals in Q1, Q2, Q3 and Q4, respectively. In addition, the association between NSM and colorectal polyps is shown in Table 3. In Model C, with the Q1 group as reference, the adjusted ORs for Q2, Q3 and Q4 were 1.47 (95% CI: 1.13 - 1.91, P = 0.004), 1.91 (95% CI: 1.55 - 2.37, P < 0.001) and 2.64 (95% CI: 1.91 - 3.64, P < 0.001), respectively.

**Table 3:** Prevalence and ORs of colorectal polyps among different NAFLD and smoking status in men

	Colorectal polyps	Model A	P-value	Model B	P	Model C	P-value
Q1	352/1096 (32.1%)	1	-	1	-	1	-
Q2	154/402 (38.3%)	1.31 (1.04 - 1.67)	0.025	1.49 (1.16 - 1.91)	0.002	1.47 (1.13 - 1.91)	0.004
Q3	315/691 (45.6%)	1.77 (1.46 - 2.16)	<0.001	1.88 (1.53 - 2.32)	<0.001	1.91 (1.55 - 2.37)	<0.001
Q4	113/220 (51.4%)	2.23 (1.67 - 2.99)	<0.001	2.63 (1.93 - 3.58)	<0.001	2.64 (1.91 - 3.64)	<0.001

Model A is univariate analysis.

Model B is adjusted for age and alcohol.

Model C is adjusted for age, obesity, hypertension, hyperglycemia, dyslipidemia and alcohol.

Q1: NAFLD (-)/Smoking (-); Q2: NAFLD (+)/Smoking (-); Q3: NAFLD (-)/Smoking (+); Q4: NAFLD (+)/Smoking (+).

### Stratified analysis

The analysis conducted according to the presence, type (polyp-free, adenomatous and non-adenomatous), location, size and number of colorectal polyps is shown in Table 4. In comparison with subjects in the NAFLD (-) and smoking (-) group, subjects in the NAFLD (+) and smoking (+) group had significantly increased ORs for adenomatous polyps (OR = 2.06, 95% CI: 1.38 - 3.05, P < 0.05), non-adenomatous polyps (OR = 1.97, 95% CI: 1.39 - 2.80, P < 0.05), polyps number ≥ 3 (OR = 2.05, 95% CI: 1.31 - 3.22, P < 0.05) and proximal polyps (OR = 1.58, 95% CI: 1.02 - 2.45, P < 0.05). Furthermore, individuals in the NAFLD (+) and smoking (-) group had higher ORs of adenomatous polyps (OR = 1.49, 95% CI: 1.06 - 2.09, P < 0.05), polyp size of 5 -10 mm (OR = 1.50, 95% CI: 1.03 - 2.17, P < 0.05) and bilateral polyps (OR = 1.61, 95% CI: 1.06 - 2.45, P < 0.05). Individuals in the NAFLD (-) and smoking (+) group had higher ORs for adenomatous polyps (OR = 1.33, 95% CI: 1.01 - 1.76, P < 0.05) and non-adenomatous polyps (OR = 1.86, 95% CI: 1.47 - 2.36, P < 0.05).

**Table 4:** Colonoscopic features in relation to the different NAFLD and smoking status in men

	Q1 n=1096	Q2 n=402	Q3 n=691	Q4 n=220	Q1	Q2	Q3	Q4
**Polyp-free**	744	248	376	107	1	0.68 (0.52 - 0.89)^*^	0.52 (0.42 - 0.65)^*^	0.38 (0.28 - 0.52)^*^
**Adenomaous polyps**	153	68	121	47	1	1.49 (1.06 - 2.09)^*^	1.33 (1.01 - 1.76)^*^	2.06 (1.38 - 3.05)^*^
**Non-adenomaous polyps**	199	86	194	66	1	1.23 (0.91 - 1.66)	1.86 (1.47 - 2.36)^*^	1.97 (1.39 - 2.80)^*^
**Polyp number**								
** 1**	197	79	144	50	1	1.00 (0.74 - 1.35)	0.71 (0.55 - 0.92)^*^	0.78 (0.52 - 1.15)
** 2**	64	38	68	23	1	1.38 (0.90 - 2.11)	1.06 (0.73 - 1.53)	1.28 (0.75 - 2.18)
** ≥3**	91	37	103	40	1	1.37 (0.92 - 2.04)	1.32 (0.96 - 1.82)	2.05 (1.31 - 3.22)^*^
**Size (mm)**								
** <5**	231	95	195	67	1	1.06 (0.80 - 1.40)	0.90 (0.71 - 1.13)	1.14 (0.81 - 1.61)
** 5 - 10**	90	43	94	32	1	1.50 (1.03 - 2.17)^*^	1.03 (0.74 - 1.42)	1.17 (0.72 - 1.89)
** ≥10**	31	16	26	14	1	1.04 (0.54 - 1.99)	0.83 (0.48 - 1.42)	1.35 (0.64 - 2.84)
**Location**								
** Proximal polyps**	117	40	89	30	1	1.29 (0.89 - 1.87)	0.88 (0.64 - 1.21)	1.58 (1.02 - 2.45)^*^
** Distal polyps**	173	75	146	52	1	0.93 (0.68 - 1.26)	0.88 (0.68 - 1.13)	0.80 (0.54 - 1.19)
** Bilateral polyps**	62	39	80	31	1	1.61 (1.06 - 2.45)^*^	1.10 (0.77 - 1.57)	1.50 (0.88 - 2.55)

Adjusted for age, obesity, hypertension, hyperglycemia, dyslipidemia and alcohol.

Q1: NAFLD (-)/Smoking (-); Q2: NAFLD (+)/Smoking (-); Q3: NAFLD (-)/Smoking (+); Q4: NAFLD (+)/Smoking (+).

Q1 group is as reference.

^*^: P < 0.05.

## DISCUSSION

The present study is the first to investigate the possible association of NSM and colorectal polyps. In our study, after controlling for confounding factors, the ORs for colorectal polyps in male individuals with a history of smoking and NAFLD were higher compared to individuals of the Q1 group. Furthermore, in the Q4 group, the ORs for adenomatous polyps (OR = 2.06), non-adenomatous polyps (OR = 1.97), ≥ 3 polyp number (OR = 2.05) and proximal polyps (OR = 1.58) remained higher than those in the other groups. Thus, our study demonstrated that men with NAFLD and a history of smoking have a higher risk for colorectal polyps, especially proximal polyps and multiple polyps (greater than or equal to3).

A number of previous reports have suggested that NAFLD and smoking are risk factors for colorectal adenomatous polyps and neoplasms [9, 14, 16, 17, 21]. In agreement with previous studies, our study showed significant associations between NAFLD, smoking and the presence of colorectal polyps.

Over the past two decades, many epidemiologic studies have demonstrated that smoking was associated with an increased risk for colorectal hyperplastic polyps and adenomatous polyps [9, 14]. Tobacco use is regarded as a key determinant of adenoma risk, and smoking may be important for both formation and aggressiveness of adenomas [22]. Several retrospective cohort studies have demonstrated a significant relationship between NAFLD and colorectal polyps [16, 17, 21]. The exact mechanism behind the increase in colorectal polyps induced by NSM remains unclear, but the potential factors suggested include oxidative stress, chronic inflammation and insulin resistance [8, 10–13].

Chronic inflammation is a well-known mechanism in the development of colorectal polyps. Cigarette smoking is known to increase inflammatory biomarkers, cause oxidative stress and exert pro-inflammatory effects in many organs, such as diabetic nephropathy, pancreatitis, hepatic steatosis and gastrointestinal mucosal diseases [23–26]. Furthermore, experimental studies have demonstrated that plasma inflammatory biomarkers, such as C-reactive protein, reactive oxygen species, plasma fibrinogen, interleukin-6, tumor necrosis factor-alpha and plasminogen activator inhibitor-1 are elevated in individuals with NAFLD [16, 27, 28]. These inflammatory biomarkers inhibit cell apoptosis and promote cell proliferation, angiogenesis and metastasis, which may partly explain the formation of colorectal polyps in men with NAFLD and smoking habit [16, 29, 30].

The role of insulin resistance in colorectal polyps is well recognized [31]. Cigarette smoking has been known to decrease insulin sensitivity by increasing insulin-antagonistic hormones (catecholamine, cortisol and growth hormone) [32]. Insulin resistance plays a crucial role in the pathophysiology of NAFLD and colorectal polyps [13, 18, 31]. Several experimental studies reported that insulin and the insulin-like growth factor (IGF) axis play an important role in cellular metabolism and growth [33]. The mechanism of insulin in stimulating the growth of colorectal cells has been described to be mediated by the increase in bioactive IGF-1 (which inhibits apoptosis and regulates cell cycle progression) through the regulation of hepatic growth hormone receptors and reduction of hepatic secretion of IGF binding protein-1 [34]. Smoking and NAFLD are closely related to insulin resistance, which is a risk factor for increasing the incidence of colorectal polyps [17, 22].

Our study has several limitations that need to be acknowledged. First, a high proportion of individuals undergoing health checks were office workers with a sedentary lifestyle. This particular group might differ from the general Chinese population with regard to nutritional habits and physical exercise. Therefore, a bias could have been introduced. It is possible that lifestyle habits in this population, such as consumption of high calorie, high fat diets and physical exercise might differ from those in the general population. Second, this was a single center study with a small sample size. A multicenter study with a large sample size should be conducted to further corroborate the association in the next step. Third, although the standard criterion for diagnosis of NAFLD is liver biopsy, it is too invasive for use in routine health examinations. The diagnosis of fatty liver was based on ultrasound imaging with 94% sensitivity and 84% specificity [35].

In conclusion, our study demonstrated that NSM was positively correlated with the prevalence of colorectal polyps, which should pay more attention to colonoscopy examination in their routine physical checkup.

## MATERIALS AND METHODS

### Subjects

This retrospective cross-sectional study involved individuals who were participating in a routine clinical physical examination at the Medical and Health Care Center of the First Affiliated Hospital of Wenzhou Medical University from September 2014 to November 2016. All individuals underwent physical examination, laboratory testing and an interview on lifestyle, medical history and medication use with a standard questionnaire. A total of 6035 participants (3657 males and 2378 females) were screened. Among this large study group, 3626 participants met the study exclusion criteria: incomplete intestine preparation; incomplete colonoscopic examination; female; incidence of carcinoma, inflammatory bowel disease or chronic liver disease (viral, toxic and autoimmune liver diseases); alcohol consumption >140 g/week and missing relevant data (such as tobacco use and colonoscopies). The protocol followed the tenets of the Treaty of Helsinki.

### Measurements

The clinical research coordinators collected physical assessment data, including age, sex, height, weight, smoking, alcohol use, systolic blood pressure (SBP) and diastolic blood pressure (DBP) of all subjects. At every visit, height and weight were measured by a team of trained nurses twice in the morning during health examination. While weight was measured without heavy clothing and recorded to the nearest 100 g, height was measured without shoes and recorded to the nearest mm with a stadiometer. BMI was calculated as dividing the weight (kg) by the height squared (m^2^). Blood pressure was measured with an automatic instrument (Omron, model 705cp, Kyoto, Japan) at the brachial artery of the person in a seated position after they had at least fifteen-minute rest.

Blood samples were collected and analyzed from antecubital vein sampling. Laboratory parameters including fasting plasma glucose (FPG), triglyceride (TG), total cholesterol (TC), high density lipoprotein-cholesterol (HDL-C), low density lipoprotein-cholesterol (LDL-C), alanine aminotransferase (ALT), aspartate aminotransferase (AST), alkaline phosphatase (AKP), γ-glutamyltransferase (γ-GT) and albumin (ALB) levels were subsequently analyzed by an automated analyzer (Abbott AxSYM, Park, IL). The 13 C-urea breath test was performed with an infrared spectrometer to identify *H. pylori* infection.

### Colonoscopy

Following the full bowel preparation with 4 L polyethylene glycol lavage solution, colonoscopy (OLYMPUS, Tokyo, Japan) was performed on each participant by endoscopists (graduate degrees or more) who had at least 5 years of experience in colonoscopic examination at the First Affiliated Hospital of Wenzhou Medical University. The diagnosis of colorectal polyp was established following the recommendation of the World Health Organization (WHO). A macroscopic visible protrusion from the surface of the colorectal mucosa was considered to be a colorectal polyp [36]. The examination was regarded as complete only if an endoscope reached the caecum, otherwise it was regarded as incomplete, which was excluded from the analysis. The colonoscopic data including the location, size and number of polyps was described by professional endoscopists. The location was stratified as follows: (1) Polyps in the cecum, ascending and transverse colon were defined as proximal polyps; (2) Those in the splenic flexure, rectum, descending and sigmoid colon were considered distal polyps; (3) Polyps located on both sides were called bilateral polyps. The size of the polyps was divided into three groups: less than 5 mm, 5-9 mm and 10 mm or more. The number of polyps was also divided into three groups: one, two and three or more.

### NAFLD diagnosis

Fatty liver was diagnosed by professional technicians using abdominal ultrasonography, following the guidelines for the assessment and management of NAFLD in the Asia-Pacific region [37]. NAFLD was defined as the presence of fatty liver in the absence of the following: (1) Excess alcohol consumption (>140 g/week for men or >70 g/week for women); (2) Other etiologies of liver diseases (viral hepatitis, autoimmune liver disease, schistosomiasis, drug-induced liver disease, and cholestatic or metabolic liver disease); (3) History of hepatic cirrhosis and liver carcinoma [16].

### Smoking and metabolic-related indicators

Regardless of smoking at the time of the study or former smoking habits, individuals with cessation of less than one year were defined as smokers. In addition, the definition of metabolic-related indicators were defined as follows: (1) Obesity: BMI ≥ 25 in both genders; (2) Hypertension: SBP ≥ 140 mmHg and/or DBP ≥ 90 mmHg or previously diagnosed; (3) Hyperglycemia: FPG ≥ 6.1 mmol/L or previously diagnosed; (4) Dyslipidemia: TG ≥ 2.26 mmol/L (200 mg/dL) and/or TC ≥ 6.22 mmol/L (240 mg/dL) and/or HDL-C < 1.04 mmol/L (40 mg/dL) and/or LDL-C ≥ 4.14 mmol/L (160 mg/dL) [38].

### Statistical analysis

Data analyses were performed using SPSS (SPSS version 23.0 for Windows), and continuous variables were expressed as the mean ± standard derivation (SD). Student’s t-test was used to compare significant differences between polyp-free group and colorectal polyps group. Univariate and multivariate logistic analyses were performed to determine the unadjusted association of clinical and laboratory parameters with prognosis. To further analyze the possible interactive effects of NAFLD and smoking on the prevalence of colorectal polyps in men, individuals were divided into four groups: Q1: NAFLD (-)/smoking (-); Q2: NAFLD (+)/smoking (-); Q3: NAFLD (-)/smoking (+); Q4: NAFLD (+)/smoking (+), according to the different conditions of NAFLD and smoking. Multiple logistic regression analyses were used to analyze the possible effects of NAFLD and smoking on the prevalence of colorectal polyps in men after adjustment for confounding factors including age, obesity, hypertension, hyperglycemia, dyslipidemia and alcohol use. Then, stratified analyses were also performed to determine whether the association between NSM and colorectal polyps persisted in each subgroup (adenomatous polyp, non-adenomatous polyp, number, size and location) after adjusting for confounding factors. A two-sided P value of less than 0.05 was considered statistically significant, and each odds ratio (OR) was presented together with its 95% confidence interval (CI).

## SUPPLEMENTARY MATERIALS TABLE



## Abbreviations

AKP: alkaline phosphatase
ALT: alanine aminotransferase
AST: aspartate aminotransferase
BMI: body mass index
CIs: confidence intervals
DBP: diastolic blood pressure
FPG: fasting plasma glucose
GATS: the Global Adult Tobacco Survey
γ-GT: γ-glutamyltransferase
HDL-C: high density lipoprotein-cholesterol
IGF: insulin-like growth factor
LDL-C: low density lipoprotein-cholesterol
MS: metabolic syndrome
NAFLD: non-alcoholic fatty liver disease
NASH CRN: non-alcoholic steatohepatitis clinical research network
NSM: NAFLD and smoking in men
OR: odds ratio
SBP: systolic blood pressure
SD: standard deviation
TC: total cholesterol
TG: triglyceride
WHO: World Health Organization
