# Supplementary material for: Impact of non-alcoholic fatty liver disease and smoking on colorectal polyps
Source: Oncotarget. 2017 Aug 24;8(43):74927–35. doi: 10.18632/oncotarget.20462 (PMC5650390; doi:10.18632/oncotarget.20462)
Supplement: Supplementary file 1 [file oncotarget-08-74927-s001.pdf]

Impact of non-alcoholic fatty liver disease and smoking on colorectal polyps

SUPPLEMENTARY MATERIALS

Supplementary Table 1: Baseline characteristics of different NAFLD and smoking status in men

	NAFLD and smoking			
	**Q1 (n=1096)**	**Q2 (n=402)**	**Q3 (n=691)**	**Q4 (n=220)**
**Age (years)**	47.46 ± 11.01	45.93 ± 9.26	46.70 ± 9.58	45.45 ± 9.02
**Weight (kg)**	67.70 ± 9.13	76.15 ± 8.92	67.81 ± 10.05	77.27 ± 10.19
**Height (cm)**	168.80 ± 5.76	168.98 ± 5.45	169.24 ± 5.64	169.57 ± 5.58
**BMI (kg/m^2)**	23.75 ± 2.82	26.65 ± 2.71	23.64 ± 3.03	26.84 ± 3.03
**SBP (mmHg)**	128.55 ± 17.06	131.32 ± 16.50	126.13 ± 16.85	130.93 ± 16.53
**DBP (mmHg)**	76.70 ± 11.74	79.87 ± 11.96	75.19 ± 11.82	80.07 ± 11.38
**FPG (mmol/L)**	4.79 ± 1.10	5.33 ± 1.70	4.81 ± 1.25	5.32 ± 1.89
**TG (mmol/L)**	1.74 ± 1.17	2.97 ± 3.20	1.92 ± 1.18	3.28 ± 3.27
**TC (mmol/L)**	5.29 ± 1.00	5.66 ± 1.34	5.39 ± 1.02	5.74 ± 1.59
**HDL-C (mmol/L)**	1.27 ± 0.30	1.13 ± 0.23	1.23 ± 0.28	1.08 ± 0.20
**LDL-C (mmol/L)**	3.20 ± 0.80	3.37 ± 0.92	3.27 ± 0.84	3.34 ± 0.99
**ALT (IU/L)**	28.99 ± 21.05	49.55 ± 37.42	30.64 ± 38.86	50.46 ± 28.93
**AST (IU/L)**	27.07 ± 12.50	35.72 ± 27.74	27.63 ± 18.72	35.41 ± 15.27
**AKP (IU/L)**	76.22 ± 20.30	78.55 ± 19.58	80.20 ± 25.07	83.18 ± 21.16
**γ-GT (IU/L)**	47.01 ± 51.11	80.94 ± 170.30	61.84 ± 81.14	87.80 ± 80.02
**ALB (g/L)**	45.16 ± 3.38	45.73 ± 2.80	44.70 ± 3.05	45.68 ± 3.11
**H. pylori n (%)[a]**	478 (46.2%)[a]	219 (57.5%)[a]	339 (51.9%)[a]	113 (53.8%)[a]
**Alcohol n (%)**	174 (15.9%)	74 (18.4%)	236 (34.2%)	64 (29.1%)
**Colorectal polyps n (%)**	352 (32.1%)	154 (38.3%)	315 (45.6%)	113 (51.4%)

Q1: NAFLD (-)/Smoking (-); Q2: NAFLD (+)/Smoking (-); Q3: NAFLD (-)/Smoking (+); Q4: NAFLD (+)/Smoking (+). BMI: body mass index; SBP: systolic blood pressure; DBP: diastolic blood pressure; FPG: fasting plasma glucose; TG: triglyceride; TC: total cholesterol; HDL-C: high density lipoprotein-cholesterol; LDL-C: low density lipoprotein-cholesterol; ALT: alanine aminotransferase; AST: aspartate aminotransferase; AKP: alkaline phosphatase; γ-GT: γ-Glutamyltransferase; ALB: albumin; H.pylori: helicobacter pylori.
[a]: data are extracted from 2278 subjects.
